# The relationship between subjective perception and the psychological effects of patients in spatial isolation

**DOI:** 10.3205/dgkh000296

**Published:** 2017-08-10

**Authors:** Fabienne Ibert, Monika Eckstein, Frank Günther, Nico T. Mutters

**Affiliations:** 1 Heidelberg University Hospital, Institute of Medical Psychology in the Center for Psychosocial Medicine, Heidelberg, Germany; 2 Heidelberg University Hospital, Department of Infectious Diseases, Heidelberg, Germany

**Keywords:** spatial isolation, psychological effects, anxiety, infection control

## Abstract

**Background:** Spatial isolation is a common infection control measure, but negative psychological effects are often neglected. We investigated which factors influence the perception of single room isolated patients.

**Methods:** In the present correlative cross-sectional study, 32 isolated patients have been interviewed within three departments of the Heidelberg University Hospital, one of Germany’s largest hospitals. The following questionnaires were used: 10-Item Big Five Inventory (BFI-10), Positive and Negative Affect Schedule (PANAS), Hospital Anxiety and Depression Scale (HADS) and a self-developed questionnaire to evaluate the individual experience of isolation. Data were analysed using correlation and regression analysis.

**Results:** A significant positive correlation was found between the isolation period and anxiety (r=.42, p<.05). Interestingly, a significant positive correlation was demonstrated between the duration of contact to nursing staff and negative daydreaming (r=.89, p<.01). The activity watching television was associated with higher levels of anxiety (r=.38, p<.05). Surfing the internet had a positive relationship with thinking about beautiful things (r=.41, p<.05).

**Conclusions:** Our study results have implications how to improve the psychological situation of patients during spatial isolation. Contact between nursing staff and patients is crucial, since this contact significantly associated with negative daydreaming, probably due to increased neediness of emotional and physical care in some patients. The duration of the isolation has an influence on the experience of anxiety. Activities to cope with the isolation, however, not always have positive effects on the well-being of the patient.

## Zusammenfassung

**Hintergrund:** Die räumliche Isolierung ist eine häufig angewendete krankenhaushygienische Maßnahme. Negative psychologische Effekte werden jedoch oftmals ausgeblendet. Die vorliegende Studie untersucht, welche Faktoren die individuelle Wahrnehmung der Isolierung im Einzelzimmer beeinflussen.

**Methoden:** Im Rahmen einer korrelativen Querschnittsuntersuchung wurden bei 32 isolierten Patienten des Heidelberger Universitätsklinikums die Fragebögen 10-Item Big Five Inventory (BFI-10), Positive and Negative Affect Schedule (PANAS), Hospital Anxiety and Depression Scale (HADS) sowie ein selbsterstellter Fragebogen zum Erleben der Isolierung erhoben. Die Daten wurden mittels Korrelations- und Regressionsanalyse ausgewertet.

**Ergebnisse:** Es gab zwischen der Isolierungsdauer und Angst einen signifikanten positiven Zusammenhang (r=.42, p<.05). Auch ließ sich zwischen der Dauer des Kontakts zum Pflegepersonal und negativen Tagträumen ein signifikant positiver Zusammenhang nachweisen (r=.89, p<.01). Die Tätigkeit Fernsehen stand im Zusammenhang mit höheren Angstwerten (r=.38, p<.05). Internet surfen hatte eine positive Korrelation zu Nachdenken über schöne Dinge (r=.41, p<.05).

**Fazit:** Aus den Ergebnissen lässt sich ableiten, wie die psychologische Situation der Patienten während des Krankenhausaufenthalts verbessert werden kann. Der Kontakt zwischen Pflegepersonal und Patient spielt dabei eine bedeutende Rolle, da dieser Kontakt signifikant mit negativen Tagträumen in Zusammenhang steht. Dieser Zusammenhang erklärt sich wahrscheinlich durch die emotionale und physische Bedürftigkeit bei einigen Patienten. Ebenso hat die Dauer der Isolierung einen Einfluss auf das Angsterleben. Auch die Tätigkeiten während der Isolierung, wie Fernsehen und im Internet surfen, wirken auf das Befinden der Patienten ein, jedoch nicht immer positiv.

## Introduction

Spatial isolation is a common infection control measure, however, the psychological effects of isolation are often neglected. Patients are often isolated due to colonization with multidrug-resistant organisms (MDROs), which are important causes of healthcare-associated infections (HAI), resulting in significant mortality and associated increased healthcare costs [1]. Especially high-risk patients, thus e.g. immunocompromised or transplant patients, who are vulnerable to infections, need to be carefully protected against HAIs. One infection control measure is the isolation of colonized patients, either as spatial isolation in single rooms or as cohorting in multi-bed rooms. Nevertheless, especially the spatial isolation in single rooms is controversial, because it is associated with high costs and possible negative clinical outcome. Some studies have indicated that isolation has negative psychological effects on patients such as anxiety, depression and a deterioration of quality of care [2]. Nevertheless, isolation is a confirmed and important infection control measure used in hospitals. Therefore, the impact of the psychological effects must be examined thoroughly. Measures to reduce those have to be developed. There are hardly any studies on this topic and the few which are published report controversial results. On the one hand, some studies have found the isolation to be stressful [3], [4]. On the other hand other studies failed to show negative psychological effects [5]. The spatial isolation and activities during this time probably could have effects on anxiety and depression characteristics of the isolated patients, both in positive and negative ways. The aim of this study was to assess the individual perception of isolation to gain more insights into the experience of isolation and the resulting psychological effects.

## Methods

### Study design and setting

The study was conducted as a correlational cross-sectional study. The analysed data are based on single interviews, without experimental manipulation and correlations between experience and the psychological effects in hospitalized isolated patients. The interviews were performed in three departments (internal medicine, orthopaedics, tuberculosis centre) of the Heidelberg University Hospital, a 2,200 bed tertiary care university teaching hospital and one of the largest and most renowned hospitals in Germany. Spatial isolation was defined as housing in single rooms or blocking of neighbouring beds in multi-bed rooms due to MDRO carriage or tuberculosis infection, respectively. Additional infection control measures included the use of gloves, gowns, and masks (tuberculosis) when entering the room. Informed consent was obtained prior to the interviews. 

### Participants

Patients were eligible for study inclusion if they were >18 years and had been treated in isolation. Patients were excluded if the healthcare professionals responsible for their care considered them to be too severely ill or if they did not speak German. Participants were 13 women and 19 men within 22–75 years (mean 51 years). The reasons for hospital admission included: malignant disease (*N*=16), gastrointestinal disease (*N*=8), respiratory disease (*N*=5) and other diseases (*N*=3). At the moment of the survey, participants had been isolated between one and 600 days (mean=62.5, standard deviation (SD) ±140.2).

### Questionnaire

Symptoms of anxiety and depression were assessed using the validated Hospital Anxiety and Depression Scale (HADS) [6]. To measure affects, the Positive and Negative Affect Schedule (PANAS) was used [7]. To assess how the patients experience the isolation a self-developed qualitative questionnaire was used. The patients were asked to indicate the hours per day they spend engaging in activities during the isolation. Possible activities were doctor’s visit, contact to nursing staff, watching television, telephone conservations, receiving visitors, daydreaming, reflecting, reading, online activities, and writing something. They were also asked to indicate what they considered helpful and what they perceived as a burden. 

### Statistical analysis

For descriptive purposes, arithmetic mean value, standard deviation, median interquartile range, and cumulative frequencies were calculated. The Pearson correlation test was used to show the coherences between the experience in the isolation and the psychological effects. Linear regression analysis was used to test the prophecy of variables. P values of ≤0.05 were regarded as statistically significant. Analyses were performed using IBM SPSS Statistics 23. 

## Results

Overall, 4.16% of all patients were isolated within in the study period at the Heidelberg University Hospital. Thirty-two patients agreed to participate in the study (61.5% response rate). The average perceived doctor’s visiting time amounted 20 minutes and the contact to the nursing staff 1.5 hours. On average the patients watched TV three hours per day. They also had positive daydreams at an average of 1.5 hours per day and negative daydreams of 1.2 hours per day. The patients’ average active time in the internet was 55 minutes. The anxiety and depression scores were not clinically noticeable (Table 1 (Tab. 1)). However, the period of the isolation correlated significantly with the anxiety scores (r=.42, p=.05). The regression analysis showed that the period of isolation is a predictor for the anxiety score (R^2^= .15, p= .05). The Pearson correlation test also showed a positive correlation between the contact with nursing staff and negative daydreams (r=.89, p=.01). Negative daydream correlated with anxiety (r=.54, p=.01). Watching TV correlated with anxiety (r=.38, p=.05) and surfing in the internet with thinking about positive things (r=.41, p=.05). In the qualitative questionnaire the isolation was described in 39% (10/26) as a burden. Loneliness was five times the reason, two times the boredom, three times the protective clothing, one time the stigmatization, and four times the dependence from the nursing staff. Three patients liked the isolation, because of the single room. Three times it was stated that not the isolation is a burden, but the hospitalization. And six times the disease was named as a burden. Helpful preoccupations are listed in Table 2 (Tab. 2). Often being visited was the most helpful activity. But there was no correlation between the frequency of visits and anxiety and depression. 

## Discussion

The present study showed that anxiety and depression and also positive and negative affect scores showed high variance. On average the anxiety and depression score can be interpreted as normal. Other studies which studied patient in isolation and used the HADS showed all different average values [3], [5]. It can be assumed that these differences are related to the sample, because of differing conditions (i.e. having different healthcare workers). Other studies defined the disease and the hospitalization as the reason for the negative psychological effects [8]. These reasons were also indicated by five patients in our study. On the other side ten patients (39%) considered rather the isolation itself as a burden. We also found that a longer period of isolation correlated with higher anxiety scores. Furthermore, the patients had significantly more negative day dreams when they had more contact with the nursing staff. The potential reason could be the communication style and content of the nursing staff. That would confirm the hypothesis that the nursing staff cannot identify anxiety and depression and does not know how to react to those [9]. More frequent negative day dreams can be an indicator for communication deficits. Gammon described that isolated patients had a lower self-esteem and a lower sense of control [10]. Especially isolated patients are dependent on the nursing staff and for some it is a burden if the communication does not meet their needs. This could be the reason why isolated patients rated the contact to nursing staff in only 7.14% (2/28) as a helpful activity, while receiving visitors was rated helpful in 39.28% (11/28). Therefore, we recommend training the nursing staff in nurse-patient communication. Furthermore, negative day dreams correlated with anxiety. Thus, if patients feel misunderstood due to miscommunication with the nursing staff, risk of negative daydreaming probably increases as well, aggravating the situation.

Our data show that patients who often watched TV had higher anxiety scores, which correlated with negative thoughts. However, surfing the internet correlated with positive thoughts. We assume that the reason for these results is connected to the fact that surfing the internet requires active participation to navigate the websites. Watching TV on the other hand is very passive and does not require real participation, thus sometimes allowing patients to let their minds drift away. It occurs that patients tend to have negative thoughts while watching TV, which triggers anxiety. To fall in negative thoughts is an unconscious process, so the patient does not per se notice it and therefore might not be able to quote it. This hypothesis can explain the correlation between watching TV and higher anxiety scores. 

Unfortunately, our study has some minor limitations. One limitation of the correlational study design is that it does not allow causal interpretations. An alternative explanation might be that patients called for nursing staff due to negative daydreams or watched TV due to anxiety and surfed the internet because of their thoughts about beautiful things. Second, the information about the hours of activities in the self-developed qualitative questionnaire consists of subjective estimates by the patients. Third, our study population may not adequately represent the general hospital population. Fourth, the power was low because of the low sample size.

In summary, in this study we found potential indicators which influenced the psychological effects in isolated patients. We have confirmed the hypothesis that there is a correlation between the frequency of isolation and feelings of fear in isolated patients. In addition, we have identified potential activities in isolation which can increase or weaken psychological negative effects in isolated patients, respectively. In the present study we found a negative correlation between contact to nursing staff and the psychological well-being. The results possibly indicate deficits in the interaction and communication of nursing staff with patients. This is an issue infection control teams should be aware of when using isolation as an infection control measure. However, this association might partly be influenced by the simple fact that patients having more contact to nursing staff simply need it because they are sicker. Higher morbidity can be associated with a larger impact on the individual psychological well-being which in part could explain the found correlation. 

## Notes

### Competing interests

The authors declare that they have no competing interests relevant to this article. No funding was received to perform this study.

## Figures and Tables

**Table 1:**
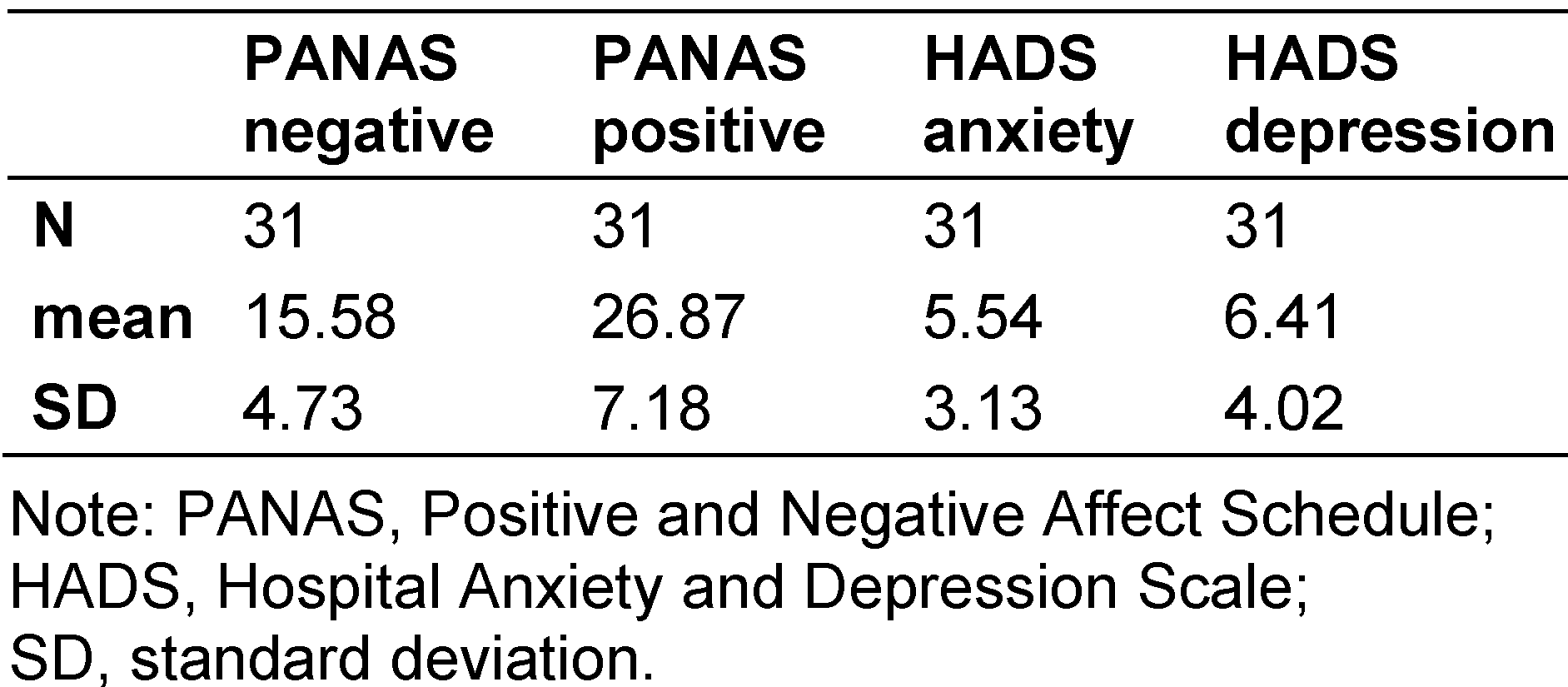
Results of the PANAS and HADS Score

**Table 2:**
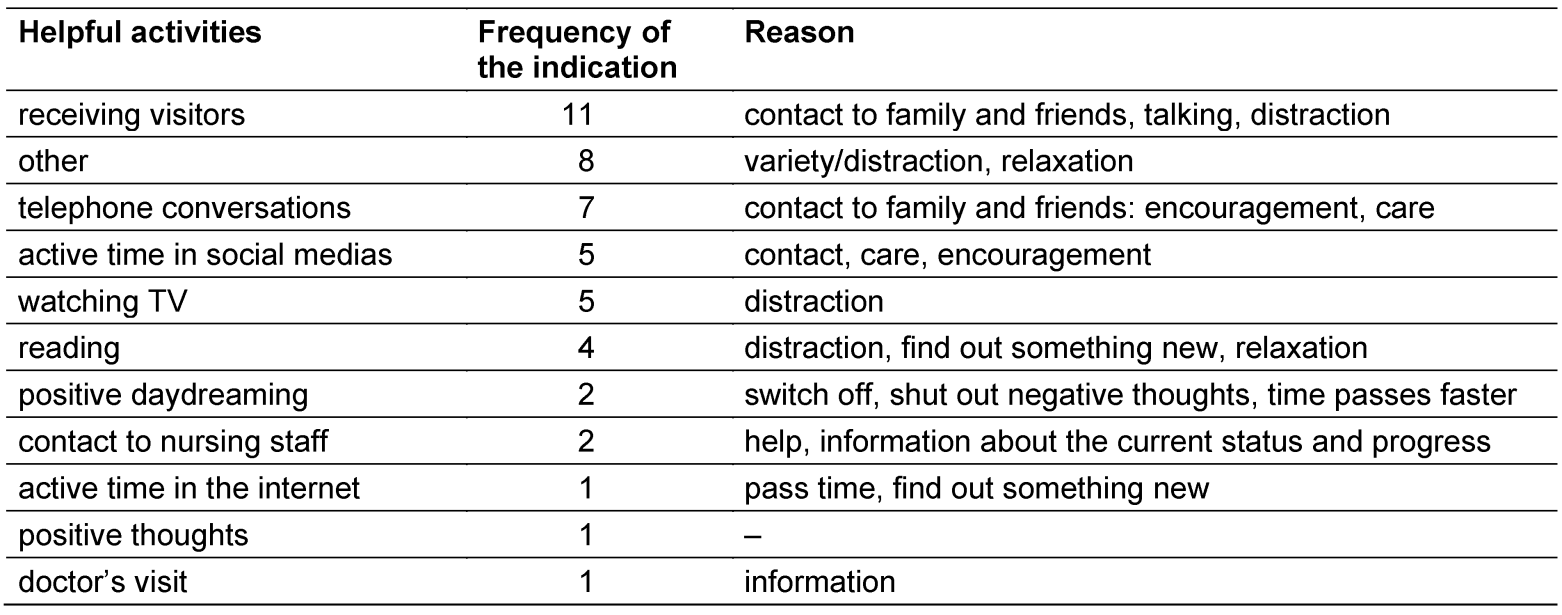
Activities that helped coping with isolation
